# Supplementary material for: Traffic trajectory data analysis technology based on HMM model map matching algorithm
Source: PLoS One. 2024 May 8;19(5):e0302656. doi: 10.1371/journal.pone.0302656 (PMC11078395; doi:10.1371/journal.pone.0302656)
Supplement: S1 Dataset — (DOC) [file pone.0302656.s001.doc]

**Figure 11(a) Comparison of accuracy of different algorithms**

| Algorithm | Parallel section | Crossing section | The overpass section | Mixed section | Average value | Standard deviations |
| --- | --- | --- | --- | --- | --- | --- |
| Direct projection algorithm | 95.2% | 93.1% | 90.4% | 84.4% | 90.8% | 0.04682 |
| Curve fitting | 96.3% | 94.9% | 94.2% | 90.8% | 94.1% | 0.02336 |
| Traditional HMM algorithm | 95.9% | 95.7% | 95.4% | 94.6% | 95.4% | 0.00572 |
| The proposed algorithm | 98.3% | 97.5% | 94.8% | 96.0% | 96.7% | 0.01559 |

**Figure 11(b) Comparison of time loss of different algorithms**

| Algorithm | Parallel section | Crossing section | The overpass section | Mixed section | Average value | Standard deviations |
| --- | --- | --- | --- | --- | --- | --- |
| Direct projection algorithm | 23.5 | 32.1 | 42.6 | 39.7 | 34.5 | 8.55195 |
| Curve fitting | 27.6 | 38.3 | 48.2 | 46.3 | 40.1 | 9.37266 |
| Traditional HMM algorithm | 53.8 | 57.5 | 68.9 | 62.8 | 60.8 | 6.56988 |
| The proposed algorithm | 53.1 | 55.9 | 60.0 | 58.8 | 57.0 | 3.09031 |
