# Supplementary material for: Traffic trajectory data analysis technology based on HMM model map matching algorithm
Source: PLoS One. 2024 May 8;19(5):e0302656. doi: 10.1371/journal.pone.0302656 (PMC11078395; doi:10.1371/journal.pone.0302656)
Supplement: Attachment — Submitted filename: Response to Reviewers Comments.docx [file pone.0302656.s002.docx]

Reviewers' comments:

Reviewer's Responses to Questions

Comments to the Author

1. Is the manuscript technically sound, and do the data support the conclusions?

The manuscript must describe a technically sound piece of scientific research with data that supports the conclusions. Experiments must have been conducted rigorously, with appropriate controls, replication, and sample sizes. The conclusions must be drawn appropriately based on the data presented.

Reviewer #1: Partly

Reviewer #2: Yes

Reply: Thank you for your comments. A map matching algorithm based on Hidden Markov Model was studied and designed to address the issue of deviation between positioning data and real roads in traffic trajectory data analysis. This algorithm not only introduces hidden Markov models, but also involves considering geometric shapes and combining time series information of orbital points. The study also explained the processing methods of trajectory data. In order to verify the performance of the algorithm designed by the research institute, experiments were conducted on the time consumption and accuracy of algorithms under different values, as well as performance comparison experiments of different algorithms. The experiments were strictly carried out according to the experimental plan. The results show that when the value is 5, the algorithm takes 51 milliseconds and has an accuracy of 95.3%, indicating better performance. Under different road conditions, the accuracy of the algorithm designed by the research institute is generally better than that of the comparative algorithms, with better performance.

In short, after revision, the manuscriptdescribed a technically sound piece of scientific research with data that supports the conclusions. Experimentshave been conducted rigorously, with appropriate controls, replication, and sample sizes. The conclusions was drawn appropriately based on the data presented.

2. Has the statistical analysis been performed appropriately and rigorously?

Reviewer #1: No

Reviewer #2: Yes

Reply: Thank you for your comments, the statistical analysis has been performed. See the details below:

In the experimental verification, the study used GPS trajectory data from the real world and employed screening methods and mapping formulas. In addition, the study also calculated the trajectory segments and generated corresponding state matrix results. The experimental data is sourced from the Gaia Trajectory Data Open Program, which selected local data from Chengdu Second Ring Road and preprocessed the data to remove redundant and missing data.

In this algorithm, the study involvedselecting the nearest line, which requires a manually specified value. 10 to 20 independent experiments were performed for each$K$ value to ensure the reliability of the statistical results. This study was measured from the time and accuracy, and providedthe appropriate value that not only guarantees accuracy, but also guarantees a certain level of efficiency. The experimental results are shown in Table 8. When $K$=5, the algorithm accuracy reached95.3, and the calculation time was only 51ms. Compared with $K$=7 (accuracy was 97.1, calculation was102ms), the calculation time was greatly reduced while maintaining high accuracy. This balance was based on consideration of the application scenarios of the algorithm.

3. Have the authors made all data underlying the findings in their manuscript fully available?

The PLOS Data policy requires authors to make all data underlying the findings described in their manuscript fully available without restriction, with rare exception (please refer to the Data Availability Statement in the manuscript PDF file). The data should be provided as part of the manuscript or its supporting information, or deposited to a public repository. For example, in addition to summary statistics, the data points behind means, medians and variance measures should be available. If there are restrictions on publicly sharing data—e.g. participant privacy or use of data from a third party—those must be specified.

Reviewer #1: No

Reviewer #2: No

Reply: Thank you for your comments. All data underlying the findings in our manuscript are fully available. See some description as follows:

The experimental data is sourced from the Gaia Trajectory Data Open Program, which selected local data from Chengdu Second Ring Road and preprocessed the data to remove redundant and missing data.

The trajectory data after removing noise is local data from the Chengdu Second Ring Road in the preprocessed Gaia trajectory data open plan.

4. Is the manuscript presented in an intelligible fashion and written in standard English?

PLOS ONE does not copyedit accepted manuscripts, so the language in submitted articles must be clear, correct, and unambiguous. Any typographical or grammatical errors should be corrected at revision, so please note any specific errors here.

Reviewer #1: No

Reviewer #2: Yes

Reply: Thank you for your comments, after revision, the manuscript is presented in an intelligible fashion and written in standard English.

5. Review Comments to the Author

Please use the space provided to explain your answers to the questions above. You may also include additional comments for the author, including concerns about dual publication, research ethics, or publication ethics. (Please upload your review as an attachment if it exceeds 20,000 characters)

Reviewer #1: The authors have submitted the manuscript " Traffic Trajectory Data Analysis and Key Technologies of Map Matching Algorithm Based on HMM Model". Although the manuscript can benefit the readers of the journal, but there are many issues with the manuscript in its current form. Some of them have been mentioned below:

1. The manuscript requires English editing for the audience to comprehend the text. There are many issue with writing of this manuscript. I would suggest the authors to ask native English speaker/editor to correct and proofread the manuscript.

Reply: Thank you for your comments. The authors have invited an native English speaker to improved the English of this paper.

2. I think the authors must revisit and revise the title, it should be focussing on Map matching algorithm using HMM model

Reply: Thank you for your comments. The title of the manuscript has been changed to “Traffic Trajectory Data Analysis Technology Based on HMM Model Map Matching Algorithm”.

In addition, some sections’ title have been revised. See below:

3. Methodology

3.1 Design of HMM and trajectory matching

3.2 Design of map matching algorithm based on the HMM

3.3 Source of trajectory data information

4. Results

4.1 Trajectory data results and analysis of HMM-based map matching algorithm

3. The formation of sentences is complex, so must be simplified. There are many instances of it, Point 1 will help authors. The first 4-5 lines in abstract needs to be rewritten.

Reply: Thanks for your comments, the mentioned sentence was changed to:

However, there are some problems in the current application scenarios, such as the deviation between positioning data and real roads, and the low accuracy of existing trajectory data traffic prediction models. Therefore, this study proposes a map matching algorithm based on hidden Markov models. This algorithm starts from the global route, selects K nearest candidate paths, and identifies candidate points through the candidate paths.

4. Section Introduction needs to re-written to add the required references. The statements has been made without proper citations. For example ... Traditional map matching algorithms....

Words are connected and needs to separated.

Reply:Thank you for your comments, the Introduction section has been re-written and citations have been added, and the connected words have been separated.

Traditional map matching (TMM) algorithms, primarily relying on geometric proximity, excel in simplicity and computational efficiency [1].They perform well in scenarios with high-quality data and simple road network structures, where geometric factors are sufficient to determine the correct path [2-3].However, these methods fall short in complex urban environments where dense road networks and frequent intersections introduce ambiguity [4-5].The conventional approach also fails to consider the temporal sequence of the trajectory data, which can lead to erroneous matches when vehicles stop or engage in complex maneuvers. In contrast, the Hidden Markov model (HMM)-based map matching algorithm offers a robust alternative by incorporating not only the geometric data but also the temporal sequence and spatial topology of the road network.By modeling the problem as a state sequence estimation, the HMM approach accounts for the probability of the vehicle's transition from one road segment to another, considering factors such as speed and direction changes, which are often neglected in traditional algorithms.This probabilistic model is particularly adept at handling the noise and imprecision inherent in GPS data, providing a more accurate reflection of the actual vehicle path [6-7].Moreover, the HMM-based method can effectively discern the correct path in scenarios where traditional methods are likely to misidentify, such as in areas with closely spaced parallel roads or intricate junctions.It can also accommodate the complexity of traffic patterns, including variable speeds and irregular movements, which are common in urban settings. Therefore, by integrating a more nuanced understanding of vehicle movement and road network dynamics, the HMM-based map matching algorithm is well-equipped to resolve the practical limitations encountered by traditional methods.

[1]Z. Lu, J. Wang, Z. Li, S. Chen, and F. Wu. "A Resource-Efficient Pipelined Architecture for Real-Time Semi-Global Stereo Matching." IEEE Transactions on Circuits and Systems for Video Technology, vol. 32, no. 2, pp. 660-673, Feb. 2022. DOI: 10.1109/TCSVT.2021.3061704.

5. Section Related work (should be section 2) is one of the poorest in this manuscript. No sentence formation is incorrect (should be past tense) and does not seem to be connected with the flow, reference numbers are missing when citing authors names. Second last paragraph seems like random reference text, should be corrected or removed with similar context. The word, ...All in all .... is non-technical.

Reply: Thank you for your feedback.

The sentence in the Section Related Work section has been changed to the past tense, and the missing reference number when citing the author's name has been supplemented. The modified content is as follows.

Cui G et al. [12] proposed a segment-based mapping matching HMM. Firstly, GPS trajectories were divided into multiple sub-trajectories by length constraint. Then, the candidate road sequence was searched for each sub-trajectory. Finally, the HMMwas used to match the sub-trajectory with the segment sequence, and the segment sequence with the highest probability was identified. The results showedthat the effectiveness and efficiency of this method were improved. Dogramadzi M et al. [13] proposed mapping matching based on a HMM. They provided a method to significantly accelerate the transition probability calculation using high data availability instances, which has previously been a major bottleneck in the performance of mapping matching algorithms.The results showedthat the proposed model was able to reduce the running time by about 0.90 compared to the traditional HMM without a significant effect on the accuracy of the algorithm. P. Alrassy et al. [14] proposed a map matching algorithm combining traffic data and trajectory data. The algorithm combined a map matching framework based on probability and weight. The ground reality data collected by the vehicle sensor device was analyzed and compared with the commonly used off-the-shelf map matching platform. The research proved that the algorithm had strong robustness. L. Huang et al. [15] proposed a batch matching strategy model for track data sub-sequences. First, the mean and median velocity of the trajectory was estimated.To ensure that the sub-sequences to be matched are on the same road section, the speed estimation was used as a constraint to determine the sub-sequence size and the local features of the road network. Therefore, an incremental map matching algorithm was proposed. The results showedthat this model hadadvantages.

[12]G. Cui, W. Bian, and X. Wang. "Hidden Markov map matching based on trajectory segmentation with heading homogeneity." GeoInformatica, vol. 25, no. 1, pp. 179-206, Jan. 2021. DOI:10.1007/s10707-020-00429-4.

[13]M. Dogramadzi, and A. Khan. "Accelerated map matching for GPS trajectories." IEEE Transactions on Intelligent Transportation Systems, vol. 25, no. 5, pp. 4593-4602, Jan. 2021, DOI:10.1109/TITS.2020.3046375.

[14]P. Alrassy, and A. W. Smyth. "OBD-data-assisted cost-based map-matching algorithm for low-sampled telematics data in urban environments." IEEE Transactions on Intelligent Transportation Systems, vol. 23, no. 8, pp. 12094-12107, Sep. 2021, DOI:10.1109/TITS.2021.3109851.

[15]L. Huang, S. Zhao, R. Luo, R. Su, M. Sindhwani, S. K. Chan, and G. R. Dhinesh. "An incremental map matching approach with speed estimation constraints for high sampling rate vehicle trajectories." ICCA, vol. 1, no. 1, pp. 758-765, Jul. 2022. DOI:10.1109/ICCA54724.2022.9831841.

**The last paragraph of the Section Related Work section has been corrected, and the usage of "All in all" has been corrected. The modified content is as follows.**

Therefore, this study proposes a map matching algorithm based on theHMM,which introduces HMM model and time series information, comprehensively considers the semantic information of adjacent time intervals of trajectory points to infer the true location,aiming to solve problems in traffic trajectory data analysis and improve the accuracy and practicality of the map matching algorithm.

6. Third section should be Methodology that include HMM-based algorithm... Need to rewrite sentence here too. Figure 1 is actually a table. Remove duplicated statements in this section, subtitles are needs revision (should be short and crisp); Formulas are used without citations and need flow in the discussion.

Reply: Thank you for your guidance. The method has been changed to section 3, and Figure 1 has been converted into separate trajectory diagram 1 and data table 1. In addition, the title of the method is rewritten and simplified. The repeated sentence has been deleted, and the sentences that need to be rewritten have already been rewritten. Finally, the formula section also cites literature. The following is the revised title and citations:

3. Methodology

3.1 Design of HMM and trajectory matching

3.2 Design of map matching algorithm based on the HMM

3.3 Source of trajectory data information

The sentence “HMM-based algorithm... ” was changed to “Design of traffic trajectory data technology for the map matching algorithm based on the HMM”.

Table 3 Part of the original data

**Figure 1 has been converted into separate trajectory diagram 1 and data table 1, as shown below.**

The specific form is shown in Figure 1.

**Figure 1 Trajectory data diagram**

From Figure 1, it can be seen that a series of data between P1 and Pn have different trajectories and information. The longitude, dimension, and time information of a series of data between P1 and Pn are shown in Table 1.

**Table 1The longitude, dimension, and time information of a series of data between P1 and Pn**

| Data | Latitude | Longitude | Time |
| --- | --- | --- | --- |
| P1 | 104.10041 | 30.70883 | 9:24 |
| P2 | 104.10018 | 30.70847 | 9:27 |
| P3 | 104.10002 | 30.70823 | 9:30 |
| P4 | 104.09996 | 30.70811 | 9:33 |
| P5 | 104.09988 | 30.70794 | 9:36 |
| P6 | 104.09983 | 30.70788 | 9:39 |
| P7 | 104.09979 | 30.70782 | 9:42 |
| P8 | 104.09975 | 30.70766 | 9:45 |
| ...... | ...... | ...... | ...... |
| Pn | ...... | ...... | ...... |

From Table 1, it can be seen that the longitude of the data from P1 to P8 is around 30.70, the latitude is around 104.0 and 104.1, and the time is around 9:30 am.

**In addition, the references for all formulas have been added, and the modified content is shown below.**

Track data $T$ is a series of data generated in chronological order, including longitude, dimensionality, and time information, as shown in formula (1)[19].

The distribution of the model can be represented as shown in formula (2) [26].

The distribution can be written as shown in formula (4) [30].

When $z_{nk}=1$, it can represent the conditional probability at the$K$ value, as shown in formula (5) [31].

By using the pending point $p$, the point to line expression is as shown in formula (6) [35].

The first $K$ segments closest to the test point $p$are selected as candidate segments [36-37], and the projection points from the processing point $p$to each candidate segment arecalculated based on the selected candidate segments $e_{1},e_{2}\cdot\cdot\cdot$ etc., as shown in formulas (7-9) [38].

It is generally assumed that this difference follows a normal distribution and its variance is set to 1 [39-40]. The standard deviation can be determined using the method given in formula (10)[41].

It calculates the distance from each candidate point to that point, and takes the mean, as shown in formula (11) [42].

Firstly, the ratio of the distance between the processing point and its precursor candidate point to the distance between the positioning point can be determined using formula (12) [43].

This instantaneous velocity can be obtained by comparing the velocity on the line segment with the velocity at the positioning point, as shown in formula (13) [44].

This can be achieved by using formula (14) [45].

In summary, the similarity of distance, speed, and angle can be combined to calculate the overall formula, as shown in formula (15) [46].

[19]Q. Xue, K. Gao, Y. Xing, J. Lu, and X. Qu. "A Context-Aware Framework for Risky Driving Behavior Evaluation Based on Trajectory Data." IEEE Intelligent Transportation Systems Magazine, vol. 15, no. 1, pp. 70-83, May. 2023. DOI:10.1109/MITS.2021.3120279.

[26]G. Lu, D. Feng, and B. Huang. "Robust algorithm for attack detection based on time-varying hidden Markov model subject to outliers." International Journal of Adaptive Control and Signal Processing, vol. 34, no. 10, pp. 1537-1558, Oct. 2020. DOI:10.1002/acs.3163.

[30]U. C. Hasar, G. Ozturk, Y. Kaya, J. J. Barroso, and M. Ertugrul. "Simple and Accurate Electromagnetic Characterization of Omega-Class Bianisotropic Metamaterials Using the State Transition Matrix Method." IEEE Transactions on Antennas and Propagation, vol. 69, no. 10, pp. 7064-7067, Oct. 2021. DOI:10.1109/TAP.2021.3098550.

[31]S. Boone, and J. McMahon. "Directional State Transition Tensors for Capturing Dominant Nonlinear Effects in Orbital Dynamics." Journal of Guidance, Control, and Dynamics, vol. 46, no. 3, pp. 431-442, Oct. 2023. DOI: 10.2514/1.G006910.

[35]T. Wang, X. Zha, F. Chen, J. Wang, L. Lin, H. Xie, F. Lin, and F. Jiang. "Research on cutting performance of coated cutting tools by a new impact test method considering contact stress condition caused by segmented chips." Journal of manufacturing processes, vol. 68, no. 2, pp. 1569-1584, Aug. 2021. DOI:10.1016/j.jmapro.2021.07.001.

[36]R. Chen, S. Yuan, C. Ma, and Z. Feng. "Tailored hidden Markov model: A tailored hidden Markov model optimized for cellular-based map matching." IEEE Transactions on Industrial Electronics, vol. 69, no. 12, pp. 13818-13827.

[37]X. Fu, J. Zhang, and Y. Zhang. "An online map matching algorithm based on second-order hidden markov model." Journal of Advanced Transportation, vol. 1, no. 1, pp. 1-12, Jul. 2021. DOI:10.1155/2021/9993860.

[38]R. Wang, M. A. Li, Y. Sun, S. ZHANG, and Z. JIA. "A NEW GEOLOGICAL SECTION MEASURING AND MAPPING METHOD:POINT COORDINATES METHOD." Journal of Geomechanics, vol. 23, no. 6, pp. 856-863, May. 2022. DOI:10.3969/j.issn.1006-6616.2017.6.856.

[39]Z. oug, B. Li, and D. Liu. "Map-Matching Using Hidden Markov Model and Path Choice Preferences under Sparse Trajectory." Sustainability, vol. 13, no. 22, pp. 12820-12820, Nov. 2021. DOI:10.3390/su132212820.

[40]H. Zhang, Y. Luo, Q. Yu, X. Zheng, and X. Li. "Map-matching approach based on link factor and hidden Markov model." Journal of Intelligent & Fuzzy Systems, vol. 40, no. 3, pp. 5455-5471, Mar. 2021, DOI:10.3233/JIFS-202292.

[41]J. Shi, D. Luo, H. Weng, X. T. Zeng, L. Lin, H. Chu, and T. Tong. "Optimally estimating the sample standard deviation from the five-number summary." Research synthesis methods, vol. 11, no. 5, pp. 641-654, Jun. 2020. DOI:10.1002/jrsm.1429 Jun.

[42]H. Huang, J. Wei, L. Zhang, B. Wang, and S. Wang. "A Coarse Alignment Method Based on Vector Observation and Truncated Vectorized κkappa-Matrix for Underwater Vehicle." IEEE Transactions on Vehicular Technology, vol. 72, no. 3, pp. 3227-3238, Mar. 2023. DOI:10.1109/TVT.2022.3220293.

[43]H. W. Hatch, D. W. Siderius, V. K. Shen, and J. R. Errington. "Efficiency Comparison of Single-and Multiple-Macrostate Grand Canonical Ensemble Transition-Matrix Monte Carlo Simulations." The Journal of Physical Chemistry B, vol. 127, no. 13, pp. 3041-3051, Mar. 2023. DOI:10.1021/acs.jpcb.3c00613.

[44]T. Zhang, W. Zhang, and F. Deng. "Strict proof for the state transition matrix of linear discrete time-varying stochastic systems." IET Control Theory and Applications, vol. 14, no. 4, pp. 674-675, Mar. 2020. DOI:10.1049/iet-cta.2019.0510.

[45]C. Zhang, J. Qin, Q. Ma, Y. Shi, and Y. Kang. "On Containment for Linear Systems With Switching Topologies: A Novel State Transition Matrix Perspective." IEEE Transactions on Cybernetics, vol. 52, no. 2, pp. 1061-1072, May. 2022. DOI:10.1109/TCYB.2020.2991514.

[46]A. Denzel, and J. Kstner. "Hessian Matrix Update Scheme for Transition State Search Based on Gaussian Process Regression." Journal of Chemical Theory and Computation, vol. 16, no. 8, pp. 5083-5089, May. 2020. DOI:10.1021/acs.jctc.0c00348.

7 Section 3 in the manuscript should be section 4 results. Again the issue with the wording confusing statements are mentioned.

Reply: Thank you for your guidance. The manuscript section 3 has been changed to Part 4 results, **,** as shown below.

1. Introduction

2. Related works

3. Methodology

4. Results

5. Discussion

6. Conclusion

Reviewer #2: This study proposes a map matching algorithm based on hidden Markov models. The proposed algorithm starts from the global route, selects K nearest candidate paths, and identifies candidate points through the candidate paths. It generates a state transition matrix using changes in speed, angle, and other information to match trajectory points to the actual route. In this paper, a key technology of map matching algorithm is proposed. The topic is interesting. However, there are some problems, which must be solved before it is considered for publication.

1. The abstract section discusses that the algorithm used in the paper is more accurate than traditional algorithms, but the running time will increase. This section only provides a simple description but lacks data comparison so it is not convincing enough. It is suggested that a detailed analysis of the advantages and disadvantages of the two methods in the "Introduction" section be added, which will demonstrate whether the map matching algorithm based on hidden Markov models can solve problems that traditional algorithms cannot solve in practical applications.

Reply: Thank you for your suggestion. In the Introduction section, it has been added to demonstrate whether the map matching algorithm based on Hidden Markov Models can solve the problems that traditional algorithms cannot solve in practical applications. The following is the content of the changed Introduction section:

Traditional map matching (TMM) algorithms, primarily relying on geometric proximity, excel in simplicity and computational efficiency [1].They perform well in scenarios with high-quality data and simple road network structures, where geometric factors are sufficient to determine the correct path [2-3].However, these methods fall short in complex urban environments where dense road networks and frequent intersections introduce ambiguity [4-5].The conventional approach also fails to consider the temporal sequence of the trajectory data, which can lead to erroneous matches when vehicles stop or engage in complex maneuvers. In contrast, the Hidden Markov model (HMM)-based map matching algorithm offers a robust alternative by incorporating not only the geometric data but also the temporal sequence and spatial topology of the road network.By modeling the problem as a state sequence estimation, the HMM approach accounts for the probability of the vehicle's transition from one road segment to another, considering factors such as speed and direction changes, which are often neglected in traditional algorithms.This probabilistic model is particularly adept at handling the noise and imprecision inherent in GPS data, providing a more accurate reflection of the actual vehicle path [6-7].Moreover, the HMM-based method can effectively discern the correct path in scenarios where traditional methods are likely to misidentify, such as in areas with closely spaced parallel roads or intricate junctions.It can also accommodate the complexity of traffic patterns, including variable speeds and irregular movements, which are common in urban settings. Therefore, by integrating a more nuanced understanding of vehicle movement and road network dynamics, the HMM-based map matching algorithm is well-equipped to resolve the practical limitations encountered by traditional methods.

[1]Z. Lu, J. Wang, Z. Li, S. Chen, and F. Wu. "A Resource-Efficient Pipelined Architecture for Real-Time Semi-Global Stereo Matching." IEEE Transactions on Circuits and Systems for Video Technology, vol. 32, no. 2, pp. 660-673, Feb. 2022. DOI: 10.1109/TCSVT.2021.3061704.

**In addition, the data comparison in the abstract has been supplemented, and the modified content is as follows**

Under different road conditions, the accuracy of the algorithm designed by the research institute is 98.3%, 97.5%, 94.8%, and 96%, respectively. The accuracy of the algorithm based on traditional hidden Markov models is 95.9%, 95.7%, 95.4%, and 94.6%, respectively. It can be seen that the accuracy of the algorithm designed by the research institute is higher.

2. In the "HMM based map matching algorithm" section, the article mentions the need to preprocess data containing a large amount of attribute information, especially for data with information loss, redundant data, and incorrect information logic. However, no specific processing standards were mentioned. Therefore, you need to supple standards for data processing in this section.

Reply: Thank you for your guidance. Data processing standards have been added. The supplementary contents are as follows:

In the data pre-processing phase, the study first examined each record to confirm if there was any missing information. It needed topay special attention to necessary fields such as time stamps and location information, any record missing these key information will be considered invalid and excluded. For example, trajectory data points that do not have a valid timestamp or location information are removed from the data set. Then, the redundant data was processed to identify and delete all duplicate records with identical properties in the data set. This step was done efficiently by comparing the hash values of the data points. Further,it should pay attention to the accuracy of the time logic. Itwas verified that the time stamps of the track points were correctly arranged in logical order, and any track points that are out of chronological order were flagged and investigated. These points were also excluded if chronological errors could not be corrected by data verification. In addition, the positional logic was scrutinized to ensure that no physically impossible speeds or distances occur. For example, if the calculation speed between two consecutive track points is beyond the possible range, itwill check and confirm whether this is caused by a data error. If necessary, it will correct or remove it accordingly.

3. According to the K-HMM algorithm flowchart in Table 4, is the value of T here the same as the track data T in Chapter 2.2? How to determine the value of T?

Reply：Thank you for your comments. The trajectory data T mentioned in Chapter 2.2 represents a series of data generated by the chronological length. The T-value mentioned in the K-HMM algorithm flow chart in Table 4 actually refers to the number of repetitions of the experiment, which is a completely different concept from the T-value of the trajectory data. The number of repetitions T of the experiment is to ensure the reliability and statistical significance of the experimental results. Is to select the nearest K different segments, and construct a matrix, so as to calculate can express the specific line group. To improve professionalism and readability, the T here has been changed to N.

**In addition, the determination method of T value is as follows.**

The determination of trajectory data$T$ is mainly obtained through sampling instruments, such as smartphones with positioning functions, GPS positioning terminals, and Radio Frequency Identification (RFID) tag technology. The mobile internet can locate the location of the mobile phone through wireless signals, sample and record, and then connect the sampling points to form the mobile phone holder's motion trajectory data. The GPS positioning terminal will use a fixed sampling frequency to collect and record information such as longitude and latitude of the terminal's location. RFID tag technology marks objects, locates them through RFID devices, and obtains their position data information.

4. How many experiments are conducted for each K value in Table 7 to determine the time consumption and accuracy? Is it universal?

Reply: Thank you for your comments. The description of the number of experiments for each K value, as well as the determination of time and accuracy, has been added, and the following is the added content:

In this algorithm, the study involvedselecting the nearest line, which requires a manually specified value. 10 to 20 independent experiments were performed for each$K$ value to ensure the reliability of the statistical results. This study was measured from the time and accuracy, and providedthe appropriate value that not only guarantees accuracy, but also guarantees a certain level of efficiency. The experimental results are shown in Table 8. When $K$=5, the algorithm accuracy reached95.3, and the calculation time was only 51ms. Compared with $K$=7 (accuracy was 97.1, calculation was102ms), the calculation time was greatly reduced while maintaining high accuracy. This balance was based on consideration of the application scenarios of the algorithm.

5. When explaining the geometric graph matching algorithm, the paper mentions that the trajectory points based on geometric factors are located on line B, while the true matching points are on line A. This part only explains the results without describing the process.

Reply：Thank you for your guidance. This paper has supplemented the process of geometric graph matching algorithm. Here's what's been added:

This shows that if matching is based only on geometric proximity, the algorithm may ignore the logic in the time series of the trajectory points, that is, the sequence and time interval of the trajectory points. For example, A locus point may be incorrectly matched to a parallel B line because of its geometric position, when its true matching point should be on A line, especially whenthe two lines are spatially close. The complexity of urban road networks may also lead to the inaccuracy of pure geometric matching methods. Intersections, parallel roads, overpasses and other factors can make the closest distance not always represent the correct match. Therefore, a map matching algorithm considering the logic of time series was adopted in this paper.

6. In Chapter 3.1, the Figure 8(b) “Partial trajectory map” was not explicitly used in the text. Perhaps it is to express the distribution of the remaining points when 3 points are used as trajectory candidate points, but there is no clear reference in the article.

Reply: Thank you for your guidance. The explanatory content of Figure 7(b) in the revised paper "Partial trajectory chart" has been supplemented, and specific references have also been explained. The following is the supplementary content:

From Figure 7, in the process of map matching, only considering the geometric proximity of the nearest route and candidate points mightlead to matching errors. From Figure 7(a) and Figure 7(b), if candidate points were selected only based on geometric distance, then the calculated distance between the point to be processed and candidate point was closer than that of candidate point 3. Then candidate point 2 mightbe mistakenly replaced by the pre-processing point.From Figure 7(b), by adding other track points of the track data where the candidate point was located, the real candidate point of the candidate point should be candidate point 3. Therefore, in the process of map matching, only from the geometric point of view was not enough to ensure high precision results. This is similar to the research results of K. Zhang researchers. K. Zhang et al. [47] designed a spatiotemporal trajectory data compression algorithm that identifies the turning and speed change behavior of vehicles by analyzing their motion patterns, and extracts feature points from multiple perspectives, namely turning and speed change angles.

[47]K. Zhang, D. Zhao, and W. Liu. "Online vehicle trajectory compression algorithm based on motion pattern recognition." IET Intelligent Transport Systems, vol. 16, no. 8, pp. 998-1010, Aug. 2022. DOI:10.1049/itr2.12187.

6. PLOS authors have the option to publish the peer review history of their article (what does this mean?). If published, this will include your full peer review and any attached files.

If you choose “no”, your identity will remain anonymous but your review may still be made public.

Do you want your identity to be public for this peer review? For information about this choice, including consent withdrawal, please see our Privacy Policy.

Reviewer #1: Yes: Sukhjit Singh Sehra

Reviewer #2: No
