# Supplementary material for: Traffic trajectory data analysis technology based on HMM model map matching algorithm
Source: PLoS One. 2024 May 8;19(5):e0302656. doi: 10.1371/journal.pone.0302656 (PMC11078395; doi:10.1371/journal.pone.0302656)
Supplement: Attachment — Submitted filename: Response to comments.doc [file pone.0302656.s003.doc]

Reviewers' comments:

Reviewer's Responses to Questions

Comments to the Author

1. If the authors have adequately addressed your comments raised in a previous round of review and you feel that this manuscript is now acceptable for publication, you may indicate that here to bypass the “Comments to the Author” section, enter your conflict of interest statement in the “Confidential to Editor” section, and submit your "Accept" recommendation.

Reviewer #1: (No Response)

Reviewer #2: All comments have been addressed

2. Is the manuscript technically sound, and do the data support the conclusions?

The manuscript must describe a technically sound piece of scientific research with data that supports the conclusions. Experiments must have been conducted rigorously, with appropriate controls, replication, and sample sizes. The conclusions must be drawn appropriately based on the data presented.

Reviewer #1: Yes

Reviewer #2: Yes

3. Has the statistical analysis been performed appropriately and rigorously?

Reviewer #1: No

Reviewer #2: Yes

Reply: Thank you for your comments. After revision, we think the statistical analysis was performed appropriately and rigorously. Take the following revision as an example:

During the experiment, statistical analysis was also conducted. The study used chi square test to compare multiple sets of data pairwise and output a significance value *p*.

**Table 9 A table of time loss and accuracy under different Kvalues**

| / | Time loss (ms) | Accuracy rate (%) | *p* |
| --- | --- | --- | --- |
| $K=1$ | 9 | 78.7 | 0.031 |
| $K=2$ | 13 | 79.6 | 0.027 |
| $K=3$ | 33 | 85.7 | 0.023 |
| $K=4$ | 46 | 89.0 | 0.034 |
| $K=5$ | 51 | 95.3 | 0.022 |
| $K=6$ | 90 | 97.0 | 0.019 |
| $K=7$ | 102 | 97.1 | 0.017 |

*p*<0.05 in Table 9 indicates that the corresponding data difference is significant.

4. Have the authors made all data underlying the findings in their manuscript fully available?

The PLOS Data policy requires authors to make all data underlying the findings described in their manuscript fully available without restriction, with rare exception (please refer to the Data Availability Statement in the manuscript PDF file). The data should be provided as part of the manuscript or its supporting information, or deposited to a public repository. For example, in addition to summary statistics, the data points behind means, medians and variance measures should be available. If there are restrictions on publicly sharing data—e.g. participant privacy or use of data from a third party—those must be specified.

Reviewer #1: No

Reviewer #2: Yes

Reply: Thank you for your comments. The authors fully provide all the data in the manuscript. Take Figure 11 as an example:

**Figure 11 Algorithm comparison graph**

The accuracy of the algorithm designed by the research on parallel, crossing, overpass, and mixed sections was 98.3%, 97.5%, 94.8%, and 96%, respectively, with an average value of 96.65%. The accuracy of the traditional HMM algorithm was 95.9%, 95.7%, 95.4%, and 94.6%, with an average of 95.4%. The accuracy of the direct projection algorithm was 95.2%, 93.1%, 90.4%, and 84.4%, respectively, with an average value of 90.775%. The accuracy of curve fitting was 96.3%, 94.9%, 94.2%, and 90.8%, respectively, with an average value of 94.05%.

The time loss of the proposed algorithm on parallel, crossing, overpass, and mixed sections was 53.1ms, 55.9ms, 60ms, and 58.8ms, respectively, with an average value of 56.95ms. The time loss based on traditional HMM algorithm was 53.8ms, 57.5ms, 68.9ms, and 62.8ms, with an average value of 60.75ms. The time loss of the direct projection algorithm was 23.5ms, 32.1ms, 42.6ms, and 39.7ms, respectively, with an average value of 34.475ms. The time loss for curve fitting was 27.6ms, 38.3ms, 48.2ms, and 46.3ms, respectively, with an average value of 40.1ms.

5. Is the manuscript presented in an intelligible fashion and written in standard English?

PLOS ONE does not copyedit accepted manuscripts, so the language in submitted articles must be clear, correct, and unambiguous. Any typographical or grammatical errors should be corrected at revision, so please note any specific errors here.

Reviewer #1: Yes

Reviewer #2: Yes

6. Review Comments to the Author

Please use the space provided to explain your answers to the questions above. You may also include additional comments for the author, including concerns about dual publication, research ethics, or publication ethics. (Please upload your review as an attachment if it exceeds 20,000 characters)

Reviewer #1:

The authors have submitted the manuscript " Traffic Trajectory Data Analysis Technology Based on HMM Model Map Matching Algorithm". The manuscript can benefit the readers of the journal. The authors response to the corrections made in not comprehensive, how they have improved the manuscript and what sections/lines have been updated.

Reply: Thank you for your correction. The Related works section and Results section have been revised. For the Related works section, the shortcomings of other studies and the citation method have been added. For the Results section, specific data on the accuracy and time consumption of different algorithms under different road conditions have been added, and chi square tests on the time consumption and accuracy of the designed algorithms under different *K* values have been deeply discussed. In addition, *p* values have been added in Table 9.

I appreciate the authors for addressing inputs shared, but the English is still needs to be improved for making this manuscript publishable. Also, authors need to see how the related work in other articles is mentioned and how references are cited. It looks like discontinuity in the paragraphs of the manuscript. I would suggest to make the manuscript little short to say the same thing in fewer sentences.

Reply: Thank you for your feedback. The English of the manuscript has been improved, and the sentences have been re-organized. The specific development of related work in other articles has been supplemented, including research results and shortcomings. In addition, the manuscript has been streamlined. The citation method of the references in the relevant work has been modified, and the numbering of the references has been placed at the end of the sentence. Some revisions are shown as follows:

To estimate travel time from sparse or low resolution trajectory data, K. Zhang et al. designed a generative adversarial network algorithm which considered spatio-temporal correlation and generated travel time for road segments without sufficient observation data. The experimental results showed that this algorithm could effectively estimate the average travel time of the link under various data loss rates, and was superior to other algorithms. In addition, the accuracy of the algorithm reached more than 98%, and the average time was 120ms. However, the weakness of this study is that the algorithm is often not stable enough, and is prone to pattern oscillation and gradient disappearance [8]. To construct the spatial semantic road map, J. Huang et al. proposed a divide and conquer approach, which integrates multiple data sources. This method includes two tasks: road structure reconstruction and road attribute inference. Example analysis showed that in the application of Wuhan City, this method could construct a routable road map with enhanced geometric structure and rich semantic information. In addition, the spatial semantic road map constructed could provide data support for vehicle navigation and spatial data infrastructure updates, and had good quality. However, this study also has certain shortcomings, such as difficulty in data integration, data security issues, and management costs [9]. Regarding the calibration issue of the car following model, R. Keane and other researchers compared different solving algorithms and finally determined an effective optimization method. This method could calibrate any car following model to adapt to various trajectory data, including lane changes, etc. The results indicated that the quasi Newtonian algorithm using the adjoint method was faster than the genetic algorithm and achieved higher calibration model accuracy. Besides, the accuracy of the quasi Newton algorithm using adjacency method was as high as 97.52%, and the computational cost was relatively low. However, this study also has shortcomings, such as increasing the complexity of the problem, requiring a large amount of computation, and not being suitable for solving non smooth problems [10]. To generate a qualified trajectory dataset that is difficult to distinguish from real trajectories, X. Chen et al. proposed two advanced solutions, TrajGAN and TrajVAE. These methods used long short-term memory models to model trajectory features and generated trajectory data using generative adversarial networks and variational autoencoder frameworks. The experimental results showed that the average accuracy values of the TrajGAN and TrajVAE models were 97.82% and 98.87%, respectively, and the generated trajectories were very close to the existing trajectories. These methods are more accurate and stable than baseline models. However, there are also shortcomings in this study, such as the tendency for gradient vanishing, difficulty in controlling the quality of generated samples, sensitivity to parameter settings, and high demand for computing resources [11].

To solve the efficiency degradation of TMM methods based on HMM when facing dense road networks, G. Cui and other experts designed a line segment-based HMM map matching model. This model requires dividing the GPS trajectory into multiple sub-trajectories. The results indicated that the proposed method improved the effectiveness and efficiency of TMM methods. The accuracy of this method was over 92%, with an average time of 213ms and an F1 value of 0.973. However, this study also needs to be improved, such as a high demand for computing resources [12]. M. Dogramadzi and other scholars proposed a map matching algorithm based on HMM to reduce measurement errors in travel trajectory data. This algorithm could accelerate the calculation of transition probability through high data availability instances. The results showed that this model was able to reduce the running time by about 0.90 compared to the traditional HMM. The accuracy of this model could reach up to 99.21%, which can reduce the demand for processor and memory usage, and accelerate the calculation of transition probabilities. However, this study can further improve the simulation of GPS errors [13]. To solve the positioning errors in map matching, P. Alrassy and other experts proposed a map matching algorithm with traffic data and trajectory data and combined a map matching framework based on probability and weight. In addition, the ground reality data collected by the vehicle sensor device was analyzed and compared with the commonly used off-the-shelf map matching platform. The results showed that the algorithm had strong robustness, with an accuracy of 97.45%, especially when map data is dense and GPS noise is high. In addition, this algorithm can perform map matching quickly and accurately. However, this study also has certain shortcomings, such as a lack of in-depth exploration in data security and privacy, and insufficient consideration in the practical implementation [14]. To reduce the impact of positioning errors and accelerate the matching process of high sampling rate trajectories, L. Huang et al. proposed a batch matching strategy model for track data sub-sequences. To ensure that the sub-sequences to be matched are on the same road section, the speed estimation was used as a constraint to determine the sub-sequence size and the local features of the road network. The results indicated that this algorithm was superior to algorithms designed for low sampling rate trajectories. This algorithm has good efficiency and accuracy, and can effectively reduce the impact of positioning errors. However, there are still significant shortcomings in the incremental map matching algorithm, and further research is needed [15].

Reviewer #2:

(No Response)

7. PLOS authors have the option to publish the peer review history of their article (what does this mean?). If published, this will include your full peer review and any attached files.

If you choose “no”, your identity will remain anonymous but your review may still be made public.

Do you want your identity to be public for this peer review? For information about this choice, including consent withdrawal, please see our Privacy Policy.

Reviewer #1: No

Reviewer #2: No
